# Psychological impacts of Intentional Non-Medical Fentanyl Use Among People Who Use Drugs: A Systematic Review

**DOI:** 10.1192/j.eurpsy.2022.245

**Published:** 2022-09-01

**Authors:** V. Tsang

**Affiliations:** UBC - Vancouver, BC, Medicine, VANCOUVER, Canada

**Keywords:** addiction, addictive disorder, psychosis, overdose

## Abstract

**Introduction:**

The use of non-medical fentanyl and structurally related compounds has changed drastically over the last ten years. Community members working with individuals who use fentanyl intentionally currently struggle with the rapidly evolving drug markets and patterns of use, thereby failing to adapt treatment approaches and harm reduction strategies to individuals with severe opioid use disorder (OUD) and concurrent psychiatric disorders.

**Objectives:**

This systematic review aims to evaluate intentional fentanyl among PWUD by summarizing demographic variance, concurrent disorders, and resulting patterns of use.

**Methods:**

The search strategy in this study was developed with a combination of free text keywords and Mesh and non-Mesh keywords, and adapted with database-specific filters to Ovid MEDLINE, Embase, Web of Science, and PsychINFO (May 2021). The search results resulted in 4437 studies after de-duplication, of which 132 were selected for full-text review. A total of 42 articles were included in this review.

**Results:**

It was found that individuals who use fentanyl intentionally were more likely to be young, male, and Caucasian. Individuals who intentionally use fentanyl were more commonly homeless, unemployed or working illegally, and live-in cities. Independent correlates of any purposeful fentanyl use included moderate/severe depression.

**Conclusions:**

Individuals who intentionally use fentanyl are more likely to report injection drug use and polysubstance use, including cocaine use, heroin use, and methamphetamine use. Among PWUD, individuals who intentionally use fentanyl have the most severe substance use patterns, the most precarious living situation, and the most extensive overdose history and higher proportion of ever having a mental health diagnosis.

**Disclosure:**

No significant relationships.

